# Photoprotective Activity of Topsentin, A Bis(Indole) Alkaloid from the Marine Sponge *Spongosorites genitrix*, by Regulation of COX-2 and Mir-4485 Expression in UVB-Irradiated Human Keratinocyte Cells

**DOI:** 10.3390/md18020087

**Published:** 2020-01-29

**Authors:** Jiyoung Hwang, Donghwa Kim, Jae Sung Park, Hyen Joo Park, Jongheon Shin, Sang Kook Lee

**Affiliations:** College of Pharmacy, Natural Products Research Institute, Seoul National University, Seoul 151-742, Korea; jy930712@snu.ac.kr (J.H.); dkim0719@snu.ac.kr (D.K.); jaesung89@snu.ac.kr (J.S.P.); phj00@snu.ac.kr (H.J.P.); shinj@snu.ac.kr (J.S.)

**Keywords:** Topsentin, *Spongosorites genitrix*, UVB, skin inflammation, COX-2, miR-4485

## Abstract

Skin is an important barrier to protect the body from environmental stress. However, exposure to ultraviolet radiation (UV) and various environmental oxidative stresses can cause skin inflammation. Cyclooxygenase-2 (COX-2) is an inducible enzyme that mediates the formation of prostaglandin E_2_ (PGE_2_) against internal and external inflammatory stimulations. Therefore, the inhibition of COX-2 is an important approach to maintain skin health and prevent skin inflammation and carcinogenesis. Topsentin, a bis(indolyl)imidazole alkaloid isolated from the marine sponge *Spongosorites genitrix*, has been reported to exhibit anti-tumor and anti-microbial activities. However, the effect of topsentin on skin inflammation and its underlying molecular mechanism has not been elucidated. In the present study, we identified the photoprotective effects of topsentin on UVB irradiated human epidermal keratinocyte HaCaT cells. Topsentin suppresses COX-2 expression and its upstream signaling pathways, AP-1 and MAPK. Furthermore, topsentin inhibits miR-4485, a new biomarker selected from a microarray, and its target gene tumor necrosis factor alpha induced protein 2 (TNF-α IP2). The photoprotective effect of topsentin was also confirmed in a reconstructed human skin model. These findings suggest that topsentin may serve as a potential candidate for cosmetic formulations with skin inflammatory-mediated disorder.

## 1. Introduction

Skin functions as a barrier to protect the body, and it is constantly exposed to chemical and environmental stress [1]. Prolonged exposure to such stresses causes skin damage and may induce skin cancer [2]. Among various exogenous stimulants to the skin, ultraviolet (UV) radiation is reported to play a significant role in inducing photoaging, inflammation, and carcinogenesis [3,4]. There are three types of UV light: UVA (320–400 nm), UVB (290–320 nm), and UVC (100–290 nm) [5]. Of these three types of UV light, UVB is the major UV source that induces pro-inflammatory responses in the skin and causes skin DNA damage [6]. Most UVB is absorbed in the epidermis, the outermost skin layer, and the majority of the epidermis consists of keratinocytes [7]. These keratinocytes, which act as a physical barrier, have diverse receptors and can stimulate signal transduction pathways to other layers of the skin [8,9].

Cyclooxygenase (COX) is an enzyme responsible for catalyzing arachidonic acid to prostaglandins (PGs) and thromboxane. There are two forms of COX: COX-1 is constitutively expressed in the majority of tissues and organs, and COX-2 is an inducible enzyme related to the inflammatory response [10,11]. Moreover, COX-2 plays a significant role in aging and skin cancer. The activity of the COX-2 signaling pathway promotes a favorable immune environment for disease progression [12]. It is reported that a selective inhibitor of COX-2 is considered to be superior to non-selective COX inhibitors such as nonsteroidal anti-inflammatory drugs in skin aging by inhibiting caveolin-1 [13]. Therefore, there is a need to discover selective COX-2 inhibitors for the treatment of skin aging and related inflammatory diseases.

MicroRNAs (miRNAs) are small nonprotein-encoding RNAs that regulate the expression of other genes [14]. Many studies suggest that miRNAs are involved in multiple biological processes such as cell differentiation, stress resistance, and cell death [15]. In addition, various miRNAs have been identified to play significant roles in skin inflammation and disorders [16]. Moreover, the serum levels of miRNAs can be used as a biomarker for skin disease [17]. 

Topsentin is a bis(indolyl) imidazole isolated from *Spongosorites genitrix* [18]. Topsentin has been reported to exhibit anti-viral, anti-tumor, and anti-fungal activities [19,20]. However, the bioactivity of topsentin in skin disorders has not yet been studied. In this study, the COX-2 inhibitory activity of topsentin was evaluated in UVB-irradiated keratinocyte HaCaT cells. The molecular mechanisms associated with COX-2 inhibition have been elucidated. Moreover, we have identified a new miRNA, miR-4485, as a biomarker of UVB-induced inflammation in human keratinocyte HaCaT cells from a microarray. Finally, the photoprotective activity of topsentin was confirmed in a reconstructed human skin model. 

## 2. Results

### 2.1. Topsentin Inhibits UVB Induced COX-2 Protein Expression and PGE_2_ Production in Hacat Cells

**Figure 1:**
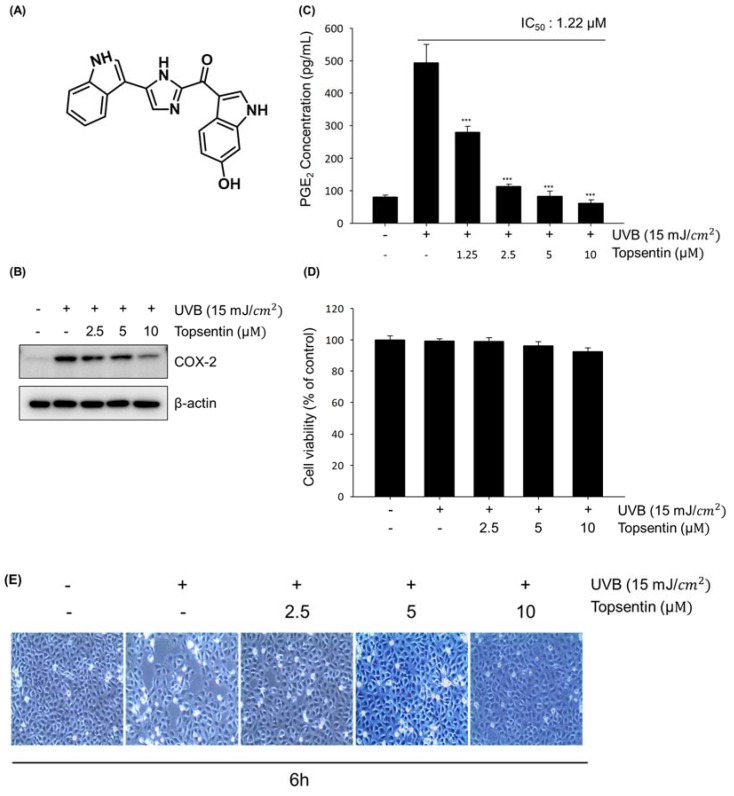
Effects of topsentin on UVB induced cyclooxygenase-2 (COX-2) protein expression and prostaglandin E_2_ (PGE2) production in HaCaT cells. (**A**) Chemical structure of topsentin. (**B**) Effect of topsentin on UVB induced COX-2 protein expression. The cells were irradiated with UVB (15 mJ/cm^2^) in the presence or absence of topsentin for 6 h. The cell lysates were analyzed by Western blotting (**C**) Effect of topsentin on UVB induced PGE2 production. The supernatants of the sample-treated cells were used to determine the PGE_2_ production. (**D**) Cell viability was determined by MTT assay with the indicated concentrations of topsentin for 6 h. (**E**) Cellular morphology was observed under a phase-contrast microscope (at 100 × magnification). *** *P* < 0.001 was considered statistically significant compared to the control group (UVB irradiated vehicle-treated cells).

To investigate the anti-inflammatory activity of topsentin (Figure 1A), HaCaT cells were treated with various concentrations of topsentin for 6 h after UVB irradiation. UVB irradiation significantly induced the COX-2 protein expression, and treatment of topsentin effectively suppressed COX-2 protein expression in a concentration-dependent manner (Figure 1B). Under the same conditions, the amount of secreted prostaglandin E_2_ (PGE_2_) was measured. Topsentin significantly inhibited the amount of secreted PGE_2_ with an IC_50_ value of 1.22 µM (Figure 1C). For evaluation of the cytotoxicity of topsentin, cell viability was measured by MTT assay (Figure 1D); it was found that topsentin did not exhibit significant cytotoxicity (cell viability of 86.6% at 10 µM). In addition, there was no remarkable change in the morphology of the cells. (Figure 1E).

### 2.2. Topsentin Suppresses UVB Induced COX-2 Gene Expression and Down-Regulates Phosphorylation of the MAPK and AP-1 Signaling Pathway

**Figure 2:**
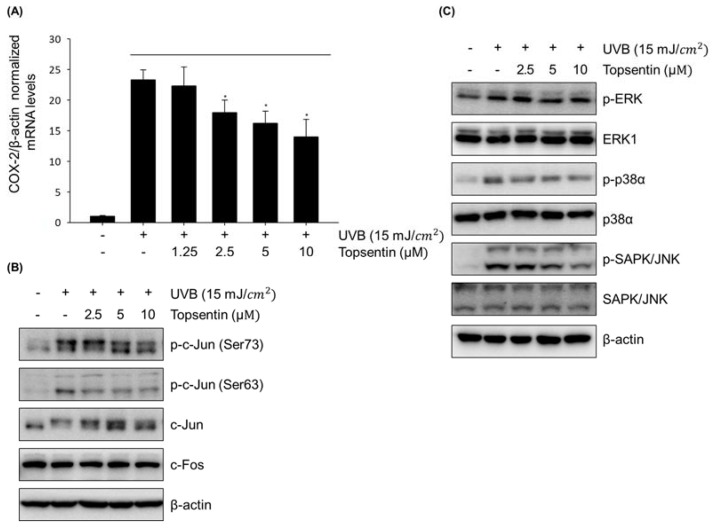
Effect of topsentin on COX-2 gene expression and its upstream signaling pathway. (**A**) Effect of topsentin on COX-2 gene expression. The cells were irradiated with UVB (15 mJ/cm^2^) in the presence or absence of topsentin for 3 h. The cell lysates were analyzed by Western blotting. (**B**) Effect of topsentin on the expression levels of AP-1 constituents. The cells were irradiated with UVB (15 mJ/cm^2^) in the presence or absence of topsentin for 0.5 h. The cell lysates were analyzed by Western blotting. (**C**) Effect of topsentin on the expression levels of MAPK constituents. The cells were irradiated with UVB (15 mJ/cm^2^) in the presence or absence of topsentin for 0.5 h. The cell lysates were analyzed by Western blotting * *P* < 0.05 was considered statistically significant compared to the control group.

To further elucidate the underlying molecular mechanisms of topsentin, we primarily investigated the gene expression of COX-2 in HaCaT cells after 3 h UVB irradiation. Topsentin significantly suppressed the UVB-induced COX-2 mRNA level in a concentration-dependent manner (Figure 2A). In particular, the mRNA level of COX-2 was 24-times higher than that of the negative control when irradiated with UVB. The treatment of topsentin (10 μM) significantly suppressed UVB-induced COX-2 mRNA expression.

Topsentin suppressed the COX-2 mRNA level generated by UVB; therefore the molecular mechanisms regulating COX-2, such as activating protein-1 (AP-1), which is composed of c-Jun c-Fos, activating transcription factor (ATF) and JDP, were analyzed. HaCaT cells treated with topsentin were collected 30 min after UVB irradiation. Topsentin inhibited the phosphorylation of c-Jun which was induced by UVB in a concentration dependent manner without affecting the protein levels of c-Jun and c-Fos (Figure 2B).

Based on these findings, we assumed that topsentin may affect the upstream pathway of AP-1, such as MAPK. As expected, topsentin effectively downregulated the phosphorylation of p38, ERK, and SAPK/JNK in a concentration-dependent manner (Figure 2C). These data suggest that topsentin is able to regulate COX-2 at the transcriptional mRNA level via the MAPK and AP-1 signaling pathway. 

### 2.3. Mir-4485 Acts as a Mediator Of UVB-Induced Skin Inflammation through Regulation of TNF-a Induced Protein 2

**Figure 3:**
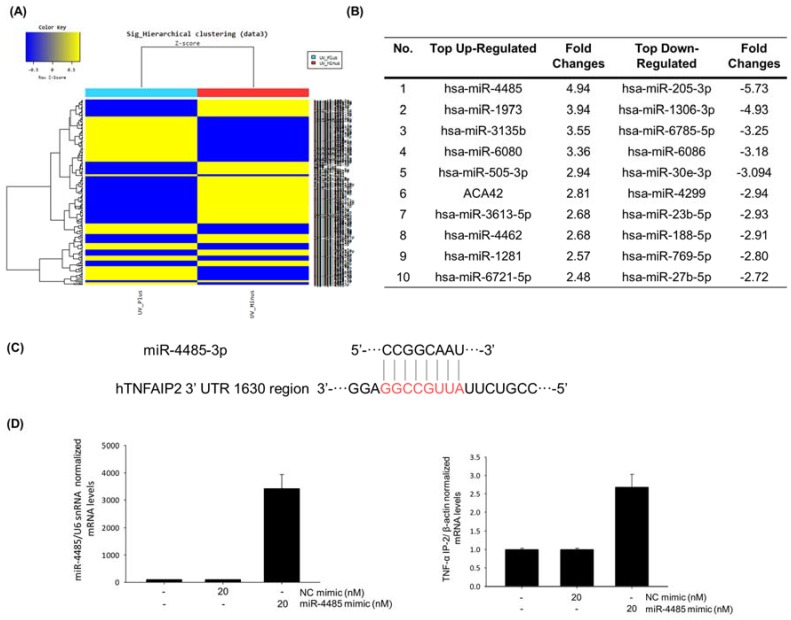
miR-4485 acts as a mediator of UVB-induced skin inflammation by regulation of tumor necrosis factor alpha induced protein 2 (TNF-α IP2). (**A**) Heatmap of miRNA expression changes by UVB irradiation obtained from microarray data. (**B**) Top 10 up- and down-regulated miRNA markers. (**C**) Sequence-based target validation of miR-4485, which was selected from Targetscan. (**D**) Target verification between miR-4485 and TNF-α IP2. Effect of miR-4485 mimic was confirmed using Real-time PCR; overexpression of miR-4485 increased the expression of TNF-α IP2.

To identify a new biomarker of skin inflammatory response caused by UVB, we analyzed the micro RNA levels of normal HaCaT cells and UVB-irradiated HaCaT cells by miRNA microarray (Figure 3A) and listed top 10 up- and down-regulated miRNAs (Figure 3B). We found that miR-4485 is the most up-regulated miRNA when irradiated with UVB, and target genes of miR-4485 were selected using miRNA TargetScanHuman 7.2 (http://www.targetscan.org/vert_72/). According to sequence-based target validation results, miR-4485 acts directly on tumor necrosis factor alpha induced protein 2 (TNF-α IP2) at the 1630 region of the 3′ untranslated region as an octamer (Figure 3C).

To verify whether miR-4485 is a genuine regulator of TNF-α IP2, we transfected a miR-4485 mimic into HaCaT cells and investigated the changes in miR-4485 level and TNF-α IP2 levels. MiR-4485 was efficiently transfected into HaCaT cells at a concentration of 20 nM, and subsequently, the TNF-α IP2 expression level was increased (Figure 3D). These findings suggest that a new biomarker, miR-4485, mediates the induction of TNF-α IP2 and following TNF-α production in UVB-irradiated HaCaT cells.

### 2.4. Topsentin Reduces TNF-a Production by Regulating miR-4485

**Figure 4:**
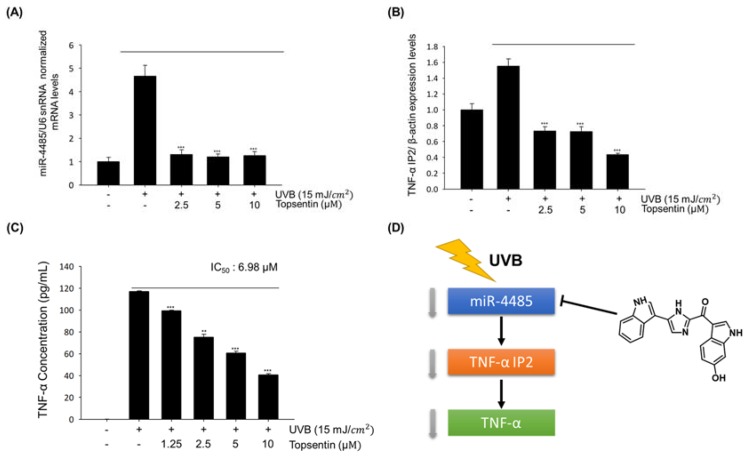
Topsentin reduces TNF-α production through the regulation of miR-4485. (**A**) Effect of topsentin on miR-4485 gene expression. The expression levels of miR-4485 induced by UVB irradiation with or without topsentin treatment were measured using real-time PCR. (**B**) Effect of topsentin on TNF-α IP2 gene expression. The expression levels of TNF-α IP2 induced by UVB irradiation with or without topsentin treatment were measured using real-time PCR. (**C**) Effect of topsentin on UVB induced TNF-α production. The supernatant of HaCaT cells was collected and analyzed by TNF-α ELISA kit. (**D**) Schematic diagram of the effects of topsentin on miR-4485 induced by UVB. ** *P* < 0.01 and *** *P* < 0.001 are considered statistically significant compared to the control group.

To further identify whether the anti-inflammatory activity of topsentin is associated with miR-4485 and of TNF-α IP2 expression, the expression levels of miR-4485 and of TNF-α IP2 by topsentin were monitored after UVB irradiation. Topsentin effectively suppressed UVB induced miR-4485 (Figure 4A) and TNF-α IP2 expression (Figure 4B) in a concentration-dependent manner.

To clarify the sequential relationship of topsentin, miR-4485, and skin inflammation, TNF-α production was measured using a TNF-α enzyme-linked immunosorbent assay (ELISA) kit. As expected, topsentin inhibited UVB-induced TNF-α production in a concentration-dependent manner with an IC_50_ value of 6.98 μM (Figure 4C). The effects of topsentin on miR-4485 induced by UVB are summarized in Figure 4D.

### 2.5. Topsentin Inhibits UVB-Induced PGE_2_ Production in a Reconstructed Human Skin Model

**Figure 5:**
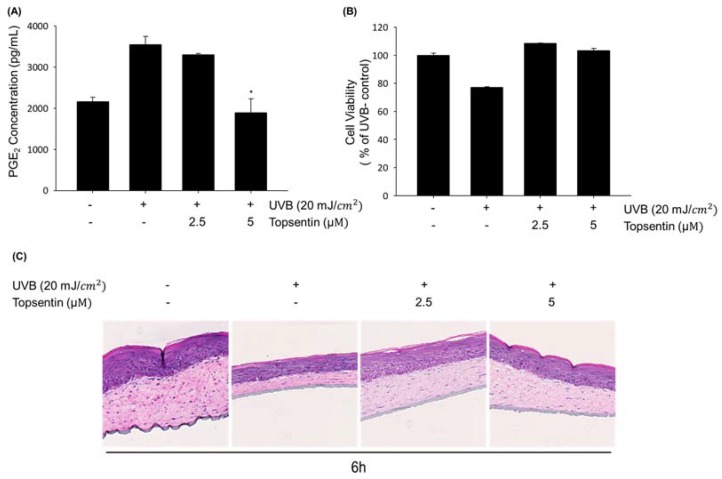
Protective effect of topsentin on UVB induced inflammation in the reconstructed human skin model, Neoderm^®^-ED. (**A**) Inhibitory effects of PGE_2_ production by topsentin on UVB-irradiated reconstructed human skin model. A reconstructed human skin model was irradiated with UVB (20 mJ/cm^2^) and the supernatant was collected after 6 h. (**B**) Effect of topsentin on the viability of the reconstructed human skin model after irradiation of UVB (20 mJ/cm^2^) for 6 h. (**C**) Effect of topsentin on UVB induced skin tissue damage. After irradiation of UVB for 6 h, skin tissues were stained by hematoxylin and eosin (H&E). * *P* < 0.01 was considered statistically significant compared to the UVB- control group.

To further investigate the effect of topsentin on UVB-induced inflammation in human skin, we employed the reconstructed human skin model Neoderm^®^-ED as a mimic of human skin. The PGE_2_ level in the supernatant of the reconstructed human skin tissue was measured in the presence or absence of topsentin by UVB irradiation. The thicknesses of both the epidermis (top) and dermis (bottom) layers of the reconstructed human skin model were found to be decreased by UVB irradiation. Treatment of topsentin effectively protected the shrinkage of the skin layers by UVB irradiation and also alleviated the PGE_2_ production in a reconstructed human skin model (Figure 5A). Cell viability was evaluated by MTT assay to determine the safety of topsentin in a reconstructed human skin model. As a result, topsentin did not show any significant cytotoxicity (Figure 5B). In addition, the hematoxylin and eosin (H&E) staining revealed that topsentin effectively restored the tissue damage induced by UVB in the layers of the reconstructed human skin model (Figure 5C).

## 3. Discussion

UV exposure is a primary cause of skin inflammation and aging, and natural product-derived compounds are often utilized in skin disease treatment and cosmetic formulations [21,22]. Skin inflammation caused by UVB is a multistep process, including COX-2 mediating PGE_2_ production and TNF-α IP-2 mediating TNF-α production [23]. COX-2, which is responsible for the formation of inflammatory lipid compound PGE_2_ and one of major enzymes in inflammation, has been investigated and reported as an important target in skin inflammation and in autoimmune diseases including arthritis [13]. Herein, we suggest COX-2 and miR-4485 as key targets for the prevention and treatment of UVB induced skin damage and inflammation. In our continuous efforts to identify anti-inflammatory agents from diverse natural product-derived compounds, we found that topsentin, a marine sponge-derived alkaloid isolated from *Spongosorites genitrix*, has a potent inhibitory activity against UVB-induced COX-2 protein expression. Further study of the anti-inflammatory activity of topsentin was investigated in UVB irradiated keratinocytes to evaluate the photoprotective effects of topsentin on photoaging by inhibiting COX-2 and miR-4485 induced by UVB.

AP-1 is a dimeric transcription factor consisting of c-Fos, c-Jun, or ATF. They can bind to AP-1 binding sites and regulate various biological functions such as cell proliferation and survival upon extracellular stimulation [24]. In keratinocyte cells, AP-1 also plays an important role in cell cycle and differentiation [25]. The transcriptional activity of AP-1 is mainly regulated by the JNK and MAPK pathway upon various stimulations [26]. Topsentin significantly suppressed the phosphorylation of the transcription factors AP-1, c-Jun, and c-Fos. Suppression of phosphorylation of c-Jun led to a reduction of target protein COX-2, a major enzyme for PGE_2_ production in keratinocytes. Additionally, the down-regulation of c-Jun phosphorylation was associated with the MAPK pathway, which is the upstream signaling pathway of AP-1. Topsentin significantly reduced the phosphorylation of MAPK constituents caused by UVB, and subsequent AP-1 phosphorylation and COX-2 expression and PGE_2_ production. Anti-inflammatory and protective effects of topsentin were further confirmed in the reconstructed human skin model composed of the epidermis (keratinocytes) and dermis (fibroblasts in the collagen matrix). The irradiation of UVB resulted in shrinkage of both skin layers which was visualized by H&E staining. Treatment with topsentin partially prevented UVB damage without affecting the viability of reconstructed skin tissues. Moreover, the production of PGE_2_ was decreased in a topsentin-treated group compared to that in the UVB-irradiated vehicle-treated control group.

Recent findings demonstrate that miRNAs play key roles in regulating skin aging and inflammation [16]. To investigate new miRNA biomarkers, we subjected normal HaCaT cells and UVB irradiated HaCaT cells to miRNA microarray and sequence-based target validation using software and miRNA mimic transfection verification. Target genes of the top 10 up- and down-regulated miRNAs were examined to find plausible miRNA regulating inflammation-related biomarkers. As a result, miR-4485 was selected as a new biomarker for UVB-induced skin inflammation because it has a complementary sequence with the TNF-α IP2 gene, which is reported to modulate TNF-α expression. MiR-4485 is reported to regulate mitochondrial functions in breast cancer cells and inhibit tumorigenicity [27]. MiRNAs are known to inhibit target genes that have complementary promoter sequences [28]. However, the present studies suggest that overexpression of miR-4485 is positively correlated with the upregulation of TNF-α IP2. Some reports suggest that complementary promoter sequences of miRNA and genes may contribute to the upregulation of gene expression [29]. Topsentin regulates the expression of the new biomarker miR-4485 in the TNF-α IP2 pathway and subsequent production of TNF-α in UVB-irradiated HaCaT cells. These findings suggest that topsentin has photoprotective effects against UVB damage in keratinocyte cells and the reconstructed human skin model.

## 4. Materials and Methods

### 4.1. Materials

The test compound topsentin (MW = 342.11, Figure 1A) was isolated from the marine sponge *Spongosorites genitrix* collected from Jeju Island, Korea on September 16, 2007, following a pre-described protocol [18]. Topsentin was dissolved in 100% DMSO and stored at −20 °C for subsequent experiments. Dimethyl sulfoxide (DMSO), bicinchoninic acid (BCA), copper sulfate, trichloroacetic acid (TCA), and thiazolyl blue tetrazolium bromide (MTT) were purchased from Sigma-Aldrich (St. Louis, MO, USA). Dulbecco’s modified Eagle medium (DMEM), fetal bovine serum (FBS), trypsin-EDTA solution (1×), and antibiotic-antimycotic solution (100×) were purchased from Invitrogen (Carlsbad, CA, USA). COX-2, β-actin, c-Fos, p-ERK, ERK, p-p38α, and p38α antibodies were purchased from Santa Cruz Biotechnology (Santa Cruz, CA, USA). C-Jun, p-c-Jun (Ser73), p-c-Jun (Ser63), p-SAPK/JNK, and SAPK/JNK antibodies were purchased from Cell Signaling Biotechnology (Danvers, MA, USA).

### 4.2. Cell Culture

HaCaT cells were purchased from American Type Culture Collection (ATCC, Manassas, VA, USA). HaCaT cells were cultured in DMEM (Gibco-Invitrogen) supplemented with 10% FBS, 100 units/mL penicillin, 100 μg/mL of streptomycin, and 250 ng/mL of amphotericin B at 37 °C in a humidified incubator under an atmosphere containing 5% CO_2_.

### 4.3. UVB Irradiation

UVB irradiation was performed as described previously [30]. Briefly, HaCaT cells were plated in 60 mm diameter dishes at a density of 22 × 10^4^ cells/well. After an additional 48 h incubation, the cells were washed with Ca^2+^- and Mg^2+^-free phosphate-buffered saline (PBS) a total of 3 times. The cell media were replaced with UV media (Colorless DMEM, Hyclone-Invirogen) with or without sample. The cells were then irradiated with 15 mJ/cm^2^ UVB using BIO-SUN (Vilber Lourmat, Marne, France). After an additional 6 h incubation, the adherent cells and supernatant samples were collected. The proteins from the cell lysates were analyzed by Western blotting analysis. The supernatant samples were analyzed using a PGE2 ELISA kit.

### 4.4. Cell Viability Assay (MTT Assay)

HaCaT cells were incubated in 500 µg/mL of MTT (Sigma-Aldrich, St. Louis, MO, USA) solution for 3 h at 37 °C. Supernatant media were removed and DMSO was added to each dish. Absorbance was measured at 570 nm, and cell viability was calculated by comparison with a control group. Cell morphology was observed and photographed on a microscope (Olympus, Tokyo, Japan).

### 4.5. Western Blotting Analysis

HaCaT cells were washed with PBS and lysed in 2× sample loading buffer (250 mM Tris-HCl; pH 6.8, 10% glycerol, 4% sodium dodecyl sulfate; SDS, 2% β-mercaptoethanol, 0.006% bromophenol blue, 5 mM sodium orthovanadate, and 50 mM sodium fluoride, Bio-Rad). The proteins were quantified by the BCA method. Equal amounts of protein (10-15 μg) were subjected to 8–12% SDS-polyacrylamide gel electrophoresis. Separated proteins were electrotransferred to polyvinylidene fluoride membranes (PVDF, Millipore, Bedford, MA, USA). The membranes were blocked with 5% bovine serum albumin (Sigma-Aldrich, St. Louis, MO, USA) for 1 h at room temperature. The membranes were then probed with specific antibodies against human COX-2, β-actin, c-Jun, phospho-c-Jun (Ser63), phospho-c-Jun (Ser73), c-Fos, ERK1, and phospho-ERK (E-4) (Santa Cruz Biotechnology, Dallas, TX, USA) and specific antibodies against human p38α, phospho-p38α (T180/Y182), SAPK/JNK, and phospho-SAPK/JNK (T183/Y185) (Cell Signaling Technology, Beverly, MA, USA). The blots were detected with an enhanced chemiluminescence (ECL) detection kit (GE Healthcare, Little Chalfont, UK) and analyzed by ImageQuant LAS 4000 (GE Healthcare).

### 4.6. RNA Isolation and Real-Time PCR

Total RNA was isolated with TRIzol reagent (Invitrogen, Carlsbad, CA, USA), and 1 μg of total RNA was reverse-transcribed using a ReverTra Ace^®^ qPCR RT Master Mix (Cat. No. FSQ-201; Toyobo Co., Okaka, Japan) according to the manufacturer’s instructions. Real-time PCR was performed using iQ SYBR Green Supermix (Bio-Rad, Hercules, CA, USA) according to the manufacturer’s instructions. The threshold cycle (C_T_) was determined using Bio-Rad CFX manager 3.1 software. Relative expression levels between compounds and untreated controls normalized to the levels of β-Actin mRNA were calculated using the comparative C_T_ method. All experiments were performed in quadruplicate, and the sequences of primers (Bioneer, Daejeon, Korea) are as follows: β-actin forward, 5′-AGC ACA ATG AAG ATC AAG AT-3′; β-actin reverse, 5′-TGT AAC GCA ACT AAG TCA TA-3′; COX-2 forward, 5′-CTT CAC GCA TCA GTT TTT CAA G-3′; and COX-2 reverse 5′-TCA CCG TAA ATA TGA TTT AAG TCA AC-3′.

### 4.7. ELISA

The accurate quantitative measurement of PGE_2_ and TNF-α secreted from cells to the supernatant level was conducted with a PGE_2_ ELISA kit (Cat. No. ab133021, abcam, Cambridge, UK) and TNF-α ELISA kit (Cat. No. 550610, BD OptEIA™, San Jose, NJ, USA) according to manufacturer’s instructions.

### 4.8. Microrna Microarray Analysis and Mirna Target Validation

The miRNA microarray expression profiles of normal HaCaT cells and UVB-treated HaCaT cells were conducted using an Illumina Human HT-12 v3 Expression BeadChip (Illumina, Inc., San Diego, CA, USA) according to the technical manual of Macrogen (Seoul, Korea). Total RNA was extracted from cells with TRI reagent (Invitrogen, Grand Island, NY, USA) following the manufacturer’s instructions. The purity and integrity of total RNA were evaluated using the Nanodrop ND-1000 spectrophotometer (NanoDrop Technologies, Wilmington, DE, USA). According to the microRNA microarray results, we performed target validation using miRNA TargetScanHuman 7.2 (http://www.targetscan.org/vert_72/) for the miRNAs highly up- and downregulated by UVB (GEO accession number: GSE142534).

### 4.9. TaqMan microRNA Assay

The expression levels of miR-4485 in HaCaT cells were determined using a TaqMan^®^ MiRNA Assay kit (Cat. No. 4427975, Applied Biosystems, Foster City, CA, USA) according to manufacturer’s instructions. Total RNA was isolated with TRIzol reagent (Invitrogen, Carlsbad, CA, USA) and then converted to miRNA using TaqMan^®^ MiRNA Assay kit (Cat. No. 4366596, Applied Biosystems) according to the manufacturer’s instructions. The specific primer for miRNA has-miR-4485-3p was used for real-time PCR to determine the miRNA expression levels, and U6 snRNA was used for normalization. All experiments were performed in quadruplicate, and the sequences of the primers were purchased from Applied Biosystems: miR-4485-3p (Assay ID: 479812), 5′-UAA CGG CCG CGG UAC CCU AA-3′; U6 snRNA (Assay ID: 001973), 5′-GTG CTC GCT TCG GCA CAT ATA CTA AAA TTG GAA CGA TAC AGA GAA GAT TAG CAT GGC CCC TGC GCA AGG ATG ACA CGC AAA TTC GTG AAG CTT CCA TAT TTT-3′.

### 4.10. Transfection of MicroRNAs

The miR-4485-3p mimic (Cat. No. SMM-002) and miRNA mimic negative control (Cat No. SMC-2002) were synthesized by Bioneer Corporation (Daejeon, Korea). The microRNA mimics were transfected into HaCaT cells by electroporation using Lipofectamine RNAiMAX (Invitrogen, CA, USA) according to the manufacturer’s instructions. The cells were transfected with 20 nM miRNAs using Lipofectamine RNAiMAX (Invitrogen, Carlsbad, CA, USA) and incubated for 24 h. The cells were then treated with the samples at the indicated concentrations for an additional 24 h.

### 4.11. Reconstructed Human Skin Model

The reconstructed human skin model Neoderm^®^-ED was purchased from Tego Science (Seoul, Korea) and analyzed according to the manufacturer’s instructions. The reconstructed human skin was irradiated with 20 mJ/cm^2^ UVB using BIO-SUN (Vilber Lourmat, Marne, France) to induce COX-2 expression and PGE_2_ production. After the treatment of 2.5 μM and 5 μM topsentin for 6 h, the supernatant sample was collected and Neoderm^®^-ED was fixed with 4% paraformaldehyde and analyzed.

### 4.12. Tissue Analysis

Reconstructed human skin (Neoderm^®^-ED) was fixed with 4% paraformaldehyde and subjected to H&E staining according to the manufacturer’s instructions. The histology of the kin tissue model was measured using Vectra (automated multimodal tissue analysis system, PerkinElmer, MA, USA).

### 4.13. Statistical Analysis

The data are presented as the mean values ± standard deviation (SD) for the indicated number of independently performed experiments. All data are representative of the results of at least three independent experiments. The statistical significance was determined using Student’s t-test or one-way analysis of variance (ANOVA) coupled with Dunnett’s t-test. Differences were considered statistically significant at * *P* < 0.05, ** *P* < 0.01, and *** *P* < 0.001.

## 5. Conclusions

In summary, we report that topsentin, a marine natural product, exhibits anti-inflammatory activity in UVB-irradiated keratinocytes through the inhibition of COX-2 expression and expression of new UVB inflammatory biomarker miR-4485.
